# Cholesterol and Hedgehog Signaling: Mutual Regulation and Beyond

**DOI:** 10.3389/fcell.2022.774291

**Published:** 2022-04-27

**Authors:** Shouying Xu, Chao Tang

**Affiliations:** National Clinical Research Center for Child Health of the Children’s Hospital, Zhejiang University School of Medicine, Hangzhou, China

**Keywords:** Hedgehog, signaling transduction, GLI, cholesterol, modification

## Abstract

The Hedgehog (HH) signaling is one of the key agents that govern the precisely regulated developmental processes of multicellular organisms in vertebrates and invertebrates. The HH pathway in the receiving cell includes Patched1, a twelve-pass transmembrane receptor, and Smoothened, a seven-transmembrane G-protein coupled receptor (GPCR), and the downstream GLI family of three transcriptional factors (GLI1-GLI3). Mutations of *HH* gene and the main components in HH signaling are also associated with numerous types of diseases. Before secretion, the HH protein undergoes post-translational cholesterol modification to gain full activity, and cholesterol is believed to be essential for proper HH signaling transduction. In addition, results from recent studies show the reciprocal effect that HH signaling functions in cholesterol metabolism as well as in cholesterol homeostasis, which provides feedback to HH pathway. Here, we hope to provide new insights into HH signaling function by discussing the role of cholesterol in HH protein maturation, secretion and HH signaling transduction, and the potential role of HH in regulation of cholesterol as well.

## Introduction

Members of Hedgehog (HH) family have been recognized as one of the major intercellular signalings involved in controlling cell growth, regulating cell survival, deciding cell fate, and patterning almost all aspects of the body plan during animal embryonic development, depending on the context. Along with establishing the patterns of cellular differentiation to direct complex organ formation, HH signaling also plays an important role in post-embryonic tissue regeneration and repair processes (Lee et al., 2016; Skoda et al., 2018; Chapouly et al., 2019; Kong et al., 2019; Salaritabar et al., 2019; Rallis et al., 2020). Dysregulation of HH signaling, including mutations, is at the root of several developmental defects, and can lead to tumorigenesis in adults as well, such as holoprosencephaly (HPE) and Gorlin’s syndrome (Abramyan, 2019; Onodera et al., 2020). The origin of the name Hedgehog comes from the phenotype of the *HH* gene mutant *Drosophila* larvae that is shorter and more spiked (Ingham, 1995). Further in 1980s, Nusslein-Volhard and Wieschaus identified mutations in the *HH* gene. Since the first isolation and cloning of *HH* gene in *Drosophila* in the early 1990s, studies have identified proteins of HH family of secreted signaling molecules in both vertebrates and invertebrates, as diverse as mammals, insects and echinoderms, although the *Caenorhabditis elegans* (round worm), one of the most famous model organisms, has no *HH* ortholog (Ingham, 1995; Kume et al., 2019; González-Méndez et al., 2020). The vertebrate homologs of the *Drosophila HH* gene can be categorized into three subgroups: Sonic Hedgehog (*SHH*), Indian Hedgehog (*IHH*), and Desert Hedgehog (*DHH*), among which *SHH* is the most broadly expressed and is thought to be responsible for the major effects on development of the craniofacial part, spinal cord, axial skeleton and limbs, while *IHH* is identified to play a major role in cartilage differentiation in the growth of long bones and affects the formation of midface and masticatory system, and *DHH* is mainly involved in the formation of mouse testis, ovary and development of nerves (Jung et al., 2015; Lopez-Rios, 2016; Liu et al., 2018; Sasai et al., 2019; Amano et al., 2020; Brooks et al., 2020; Hosoya et al., 2020; Moreau and Boucher, 2020; Ohba, 2020). In the past decades, by using *Drosophila* genetics, key components of the HH signaling pathway have been identified and a series of mechanisms have been clarified. Up to now accumulating evidence has suggested that cholesterol regulates this important signaling at many levels, without which will lead to congenital malformations and cause diseases in adults.

## Processing and Maturation of Hedgehog Protein

**FIGURE 1:**
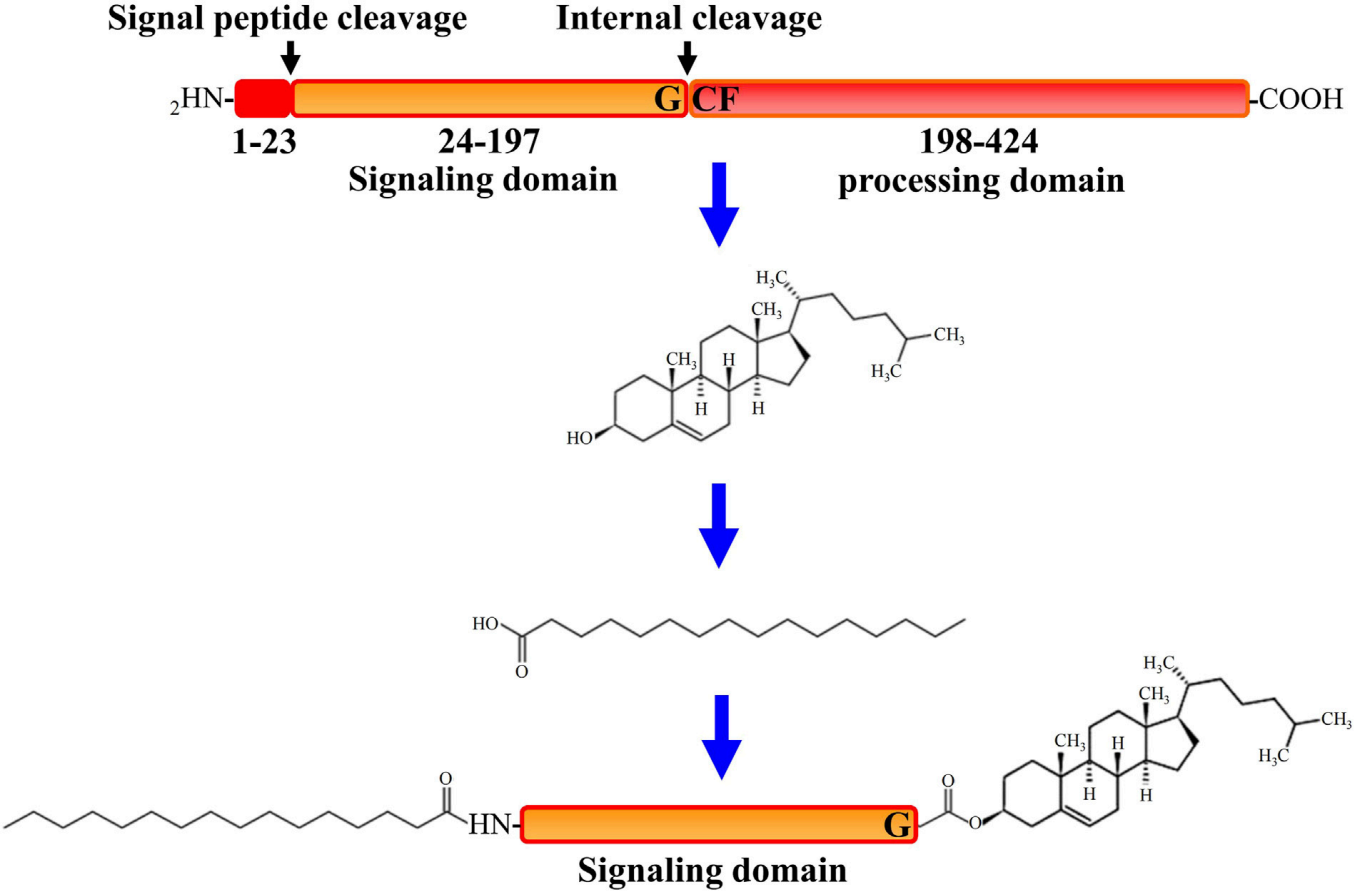
A schematic description of progress of Hedgehog protein maturation. In the first step of processing after Hedgehog (HH) protein translation, C-terminal initiates the catalytic action through its processing domain and leads to form an internal thioester between a cysteine and a glycine residue at the cleavage site. The C-terminal domain is then released by nucleophilic attack by the hydroxyl group of cholesterol, resulting in the covalent attachment of cholesterol to the C terminus of the N-terminal domain. Further more, the N terminus of the N-terminal domain is palmitoylated by the acyltransferase Skinny after signal peptide cleavage.

All HH family members share the same tertiary structure and follow the same way of maturation in HH-producing cells (Valentini et al., 1997). After translation, initially synthesized HH pro-proteins undergo multiple post-translational processing modifications to obtain activated HH proteins and subsequently to be released from the producing cells (Banavali, 2020). As depicted in Figure 1, after the signal sequence is removed, the HH molecule (∼45 kDa) first undergoes a cleavage catalyzed by its own C-terminal domain that is between the conserved glycine and cysteine residues, generating two polypeptides including the N-terminal polypeptide (HH-Np) of ∼19 kDa that contains the whole functions of the signaling in both vertebrates and invertebrates, and the C-terminal polypeptide (HH-Cp) of ∼25 kDa that has no function other than catalyzing the autoproteolytic cleavage. Mutations that block this auto proteolysis will result in impairment of HH function. During the autoproteolytic cleavage, a hydroxyl-oxygen cholesterol moiety is covalently attached to the last amino acid of the N-terminal polypeptide (HH-Np). As a result, cholesterol modified HH-Np associates tightly with the membrane, and subsequently the HH-Cp diffuses away and modified by the endoplasmic reticulum (ER) transmembrane protein acyl-transferase Skinny HH (*Ski*, also known as *sightless*, *rasp* or *central missing*, and *HHAT* in humans), a palmitic acid moiety is attached to the first amino acid of the N-terminal polypeptide (HH-Np), which is also required for HH-N activity *in vivo* (Liang et al., 2019). HH cholesteroylation is not only dispensable in the early stages of HH production and maturation, but also essential for receiving cells to receive HH signals (Manikowski et al., 2021). Thus, HH-N of full activity is with cholesteroylation at its C-terminus and palmitoylation at its N-terminus (Porter et al., 1995).

## The Hedgehog Signaling Pathway

**FIGURE 2:**
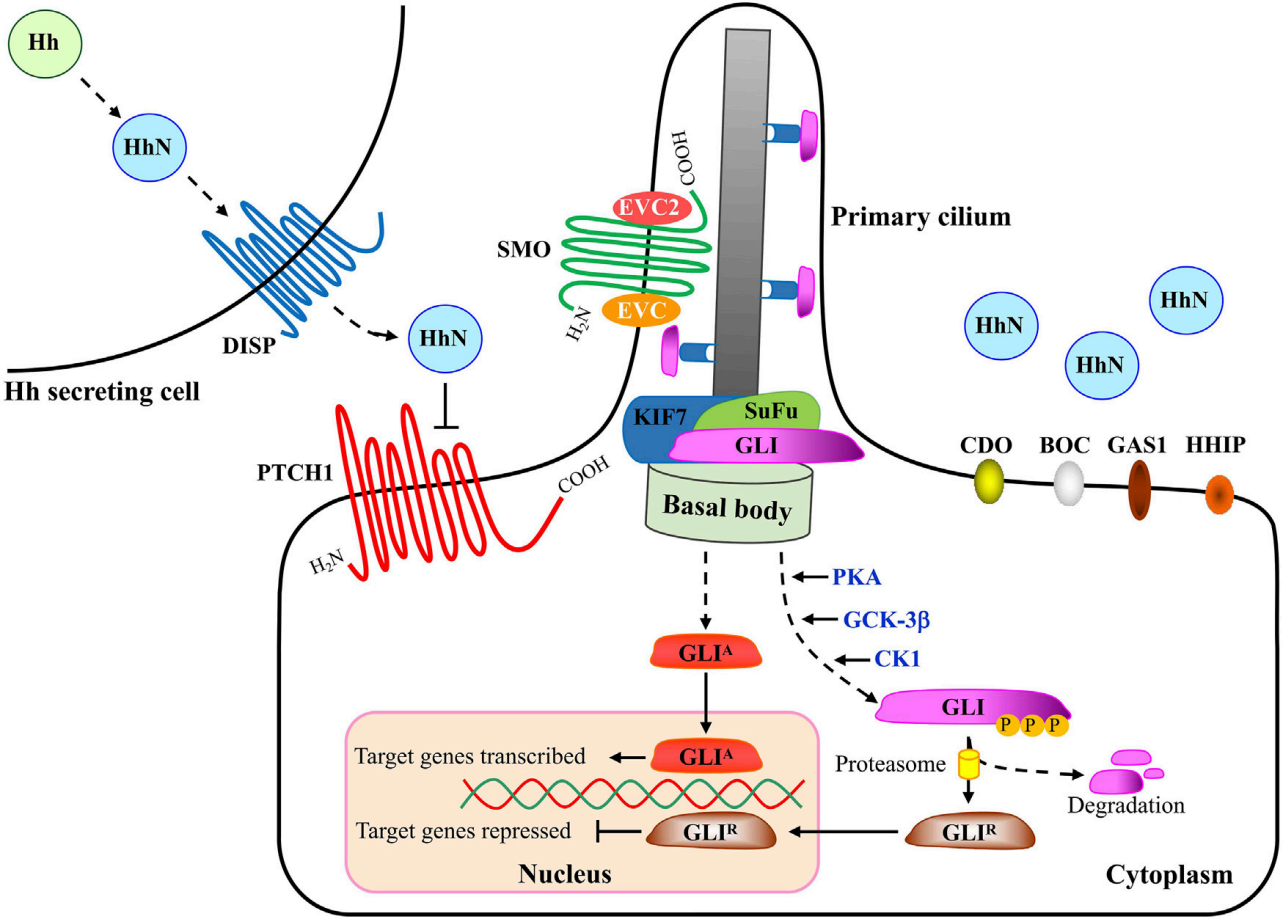
A schematic description of Hedgehog signaling transduction. When HH is absent, the 12-span-membrane receptor protein PTCH1 (Patched1) inhibits SMO (Smoothened) accumulation in the primary cilium and consequently SMO activity. SuFu can form complexes with GLI proteins and inhibit them from entering the nucleus and their transcriptional activity. Several kinases, including PKA, GSK3β and CK1 phosphorylate the C-terminal of GLIs, leading to the truncated form of full length GLI. C-terminal deleted-GLIs are thought to be strong transcriptional repressors (GLI-R) that go into the nucleus and inhibit the transcription of target genes. Upon HH ligand binding, the inhibition from PTCH1 is released and SMO is activated and translocates into the primary cilium. Within the primary cilium, SMO forms a complex with EVC and EVC2 to transduce the HH signaling response. Activated SMO leads to the release of GLIs from the SuFu complex and GLIs go into the nucleus as transcriptional activators (GLI-A) that activate the transcription of target genes. Movement of GLI proteins within the primary cilium occurs in conjunction with KIF7, a member of the kinesin family of anterograde motor proteins. Abbreviations: HH, Hedgehog; DISP, Dispatched; PTCH1, Patched1; CDO, Cell adhesion molecule-related/down-regulated by oncogenes; BOC, brother of Cdo/biregional Cdon-binding protein; Gas1, Growth arrest specific 1; HHIP, Hedgehog-interacting protein; SMO, Smoothened; EVC, Ellis van Creveld syndrome protein; EVC2, Ellis van Creveld syndrome protein 2; GLI, Glioma-associated oncogene; SuFu, Suppressor of Fused; KIF7, Kinesin family member 7.

The major components of the HH pathway are identified to be conserved in *D. melanogaster* (Briscoe and Thérond, 2013). As the first HH receptor identified, PTCH1 binds to SMO to inhibit its activity in the absence of HH ligand. At this time, the transcription factor protein GLI is distributed at the tip of primary cilium and binds to the Suppressor of Fused (SuFu) protein to form a complex, and is phosphorylated by a series of protein kinases including protein Kinase A (PKA), glycogen synthesis kinase 3β (GSK-3β) and casein kinase 1 (CK1) to form a truncated repressor. However, binding of HH to PTCH1 relieved the inhibitory effect of PTCH1 on Smo. At this time, the activated SMO is translocated to the primary cilia, and finally the transcription factor GLI is depolymerized from the GLI-SuFu complex and enters the nucleus in the form of a transcription activator (GLIA) to activate the transcription of downstream target genes (Cannonier and Sterling, 2015) (Figure 2).

In HH-responding cells, SMO is redistributed from unidentified intracellular compartments to the plasma membrane (Milenkovic et al., 2009). Activated SMO then transduces downstream signaling with the regulation of GLI transcriptional factors (Hu and Song, 2019). Three GLIs have been discovered in vertebrates, including GLI1, GLI2 and GLI3, which are in overlapping domains and are partially redundant. Similar to Ci (a 155 kDa cytoplasmic zinc finger protein) in *Drosophila*, GLI1 always acts as a transcriptional activator as it lacks the repressor domain and its expression is induced by HH ligands (Tang et al., 2015a; Infante et al., 2015; Doheny et al., 2020). Different from GLI2 that is essential for embryonic development, whose loss is embryonic lethal, GLI1 is dispensable for normal development, at least in the case of mice (Lee et al., 1997). Both GLI2 and GLI3 contain an amino-terminal repressor domain and a carboxy-terminal activator domain. In most cases GLI2 acts as a transcriptional activator (GLI-A) while GLI3 is thought to play a major role as a transcriptional repressor (GLI-R) that inhibits the expression of HH-downstream target genes. Ultimately, the balance between GLI-A and GLI-R influences HH pathway output through their accumulation and the subsequent transcription of target genes in nucleus.

In vertebrates, HH signaling is always present in primary cilia, the mechano-sensory organelles that respond to mechanical stimuli in the micro-environment. In the absence of HH signal, PTCH1 is found at the base of primary cilia, whereas GLI2 and GLI3 have been observed at the tips of the cilia and are retained in the cytoplasm in a protein complex associated with the inhibitory molecule SuFu that functions as a negative regulator in HH signaling network (Cannonier and Sterling, 2015). In addition to the well-known molecules, new members have been discovered, such as Ellis-van Creveld Syndrome (EVC) and Ellis-van Creveld Syndrome 2(EVC2), which interact with SMO and control HH signaling activity by regulating SuFu/GLI3 dissociation and GLI3 trafficking in primary cilia of chondrocytes and osteoblasts (Caparrós-Martín et al., 2013; Palencia-Campos et al., 2017). Protein kinases, including protein kinase A (PKA) and casein kinase 1 (CK1), phosphorylate SMO at several sites, leading to the increase of SMO cell-surface levels and signaling activity (Jia et al., 2004). Furthermore, a zipper-lock model was proposed in which the gradual phosphorylation at three clusters of serine residues causes a gradual conformational change in the cytoplasmic tail of Smo, thereby promoting the interaction between Smo and Costal2 in Drosophila (Fan et al., 2012). PKA, glycogen synthase kinase-3β (GSK-3β) and CK1 regulate GLI3 and a small fraction of GLI2 by phosphorylating their specific phosphor-sites in the region of C-terminus, after which, GLI3 and the small fraction of GLI2 enter the nucleus as truncated forms while most GLI2 is proteolytically degraded in the cytoplasm (Gomez et al., 2019; Yin et al., 2019). As a result, the truncated GLIs, also known as GLI repressors (GLI-R), function as repressors and suppress the transcription of HH signaling target genes. Once the canonical HH signaling is activated, GLI2 as well as GLI3 escapes from the proteolytic degradation and thereby goes into the nucleus as a full-length protein, and as transcriptional activators, the full length of GLIs activate transcription of target genes. GLIs play important roles in organ development during embryogenesis, including lungs, limbs, neural tube and craniofacial region, such as eyes, nose and teeth. Of note, during development of vertebrate neural tube, the absence of GLI2 disrupts the patterning of tissue types that are located closet to the origin of the HH signal, indicating activated GLI2 alone has the ability to induce the expression of downstream target genes of HH signaling (Minhas et al., 2015). In the nucleus, GLIs bind to the consensus and non-consensus binding sites to trans-activate or trans-inactivate their target genes. Despite the consensus GLI binding sequence “GACCACCCA”, some novel GLI binding sequences were confirmed in the past few years (Po et al., 2020), and up to now numerous target genes in HH signaling have been identified, including *Bcl2*, *Myc*, *Wnt*, *Cyclin D*, *Cyclin E* as well as *PTCH1* and *GLI1*, two members in HH pathway, which provide negative and positive feedback loops to the pathway, respectively (Ma et al., 2015; Girardet et al., 2020; Tolosa et al., 2020).

## Role of Cholesterol in Hedgehog Protein Transportation

Sterols, such as cholesterol, play a crucial role in the cell biology of all eukaryotes (Gelissen and Brown, 2017; Reboldi and Dang, 2018; Huang et al., 2020). Cellular cholesterol is derived from endogenous synthesis in the ER and from internalized lipoproteins. There is compelling evidence of the involvement of cholesterol in the proper HH signaling transduction that acts as a key regulator in both development and tumor progression (Niewiadomski et al., 2019). As a major developmental mediator in the morphogenetic field, the versatile HH molecule acts at both short and long ranges in a direct and concentration-dependent manner, which means HH family members can exert their effects not only on the cells nearby the source of the original signal but also over considerable distances (up to 30-cell diameters), at least acting as classic morphogens in some cases (Wu et al., 2019). Investigations of the past few years show that cholesterol modification affects the diffusion range of HH family members strikingly with the function to restrict the dilution and to alleviate the spread of HH ligand diffusion in the extracellular space (Dillon et al., 2003; Mateska et al., 2020; Gore et al., 2021). Although HH signaling is first thought to be a morphogen in the segmental patterning of the *Drosophila* larvae, from which a unique readout can be offered by the adult wing pattern, the importance of cholesterol modification has already been confirmed in the context of embryonic development (Purohit et al., 2020; Radhakrishnan et al., 2020). In *Drosophila*, cholesterol-unmodified HH form diffuses further away with an abnormally wide range, compared to the cholesterol-modified HH protein with a decreased affinity for plasma membrane by expressing a transgene that only encodes the HH N-terminal domain. Moreover, cholesterol-unmodified HH form does not interact properly with the Patched receptor protein, Shifted proteins and extracellular components such as heparan sulfate proteoglycan (HSPG) (The et al., 1999; Lewis et al., 2001; Dawber et al., 2005; Callejo et al., 2006; Kenwrick et al., 2019). However, recent studies indicate that cholesterol modification of HH-N is not necessary for HH-PTCH1 interaction, either functionally or structurally (Tukachinsky et al., 2016; Qi et al., 2018). In vertebrates, cholesterol modification of HH-N is essential for its function at long range, while cholesterol-unmodified HH-N only possesses the activity of short range, which was determined through a developmental ideal model system of limb bud pattern in embryonic mice. The discrepancy of conclusions between *Drosophila* and mouse possibly reflects the differences between tissues studied in each case (The et al., 1999).

Though the mechanism by which HH-Np is released from producing cells to exert long-range activity is not completely understood, one gene is identified to be involved in this process by genetic screens in *Drosophila*. This gene, *Dispatched* (*Disp*), which encodes the putative 12-pass transmembrane protein Dispatched (DISP) that contains a novel sterol-sensing domain (SSD), is found to be required for secretion of Hh-Np in *Drosophila* imaginal disc. SSD domain, first identified in proteins implicated in cholesterol homeostasis or trafficking, is required for the cholesterol-dependent regulation (Stewart et al., 2018; Cannac et al., 2020; Wierbowski et al., 2020). In *Disp* mutants, HH-Np can be hardly released to target cells or activate the target genes but accumulates in the producing cells. However, cholesterol-unmodified HH-N can be released and diffuse as it does not require DISP for secretion. Some studies have given the possible functions of DISP*,* that is, DISP is involved in the intracellular trafficking of cholesterol-modified HH-N in the secretory pathway, and once DISP reaches the cell surface it displaces cholesterol from the cell membrane (Wierbowski et al., 2020). Thus, it is likely that DISP is involved in packaging cholesterol-modified HH into freely diffusing aggregates.

In addition to the effect of cholesterol on HH protein, recent work further sheds light on another phenomenon that normal cholesterol concentrations are required for proper activation of SMO (Smoothened), the seven-transmembrane-span receptor-like protein in HH pathway, and that regulation of SMO by PTCH1 is thought to be dependent on small molecular intermediates, such as cholesterol (The et al., 1999; Callejo et al., 2006; Kenwrick et al., 2019; Dawber et al., 2005; Lewis et al., 2001; Tukachinsky et al., 2016; Qi et al., 2018; Stewart et al., 2018; Wierbowski et al., 2020; Cannac et al., 2020; Rohatgi et al., 2009; Byrne et al., 2016; Huang et al., 2016; Luchetti et al., 2016; Xiao et al., 2017; Kowatsch et al., 2019) (summarized in Table 1). The Asp 95 (D95) residue of SMO is proved to be covalently modified by cholesterol *via* an ester bond, which is enhanced by HH but inhibited by PTCH1(69). The D95N mutation of SMO is hardly modified by cholesterol and rarely localizes to the primary cilia, resulting in its failure in activation of downstream signaling transduction. Several possible mechanisms by which PTCH1 regulates SMO have been proposed, and these mechanisms are mainly focused on the primary cilium or the ciliary micro-domain: (Skoda et al., 2018) PTCH1 can regulate the overall membrane levels of cholesterol or distribution of cholesterol across the bilayer, thereby preventing cholesterol supply to the cysteine-rich domain (CRD) and/or transmembrane domain (TMD) of SMO (Zhang et al., 2018). (Kong et al., 2019) PTCH1 removes sterols from SMO CRD, thus inhibiting HH signaling (Salaritabar et al., 2019). As a sterol flippase, PTCH1 transfers sterols between the outer and the inner membrane leaflets (Deshpande et al., 2019; Kowatsch et al., 2019). More experiments are in need to address this issue.

**TABLE 1:** Summary of role of cholesterol in HH protein transportation.

References	Nature of role
The et al. (1999)	Interaction of hedgehog and HSPG requires hedgehog cholesterol modification
Callejo et al. (2006)	Hedgehog lipid modifications are required for hedgehog protein stabilization
Kenwrick et al. (2019)	Hedgehog without cholesterol modification does not interact properly with Shifted
Dawber et al. (2005)	Hedgehog without cholesterol modification has a decreased affinity for plasma membrane
Lewis et al. (2001)	Cholesterol modification of hedgehog is required for long-range signaling activity
Stewart et al. (2018)	Dispatched actively regulates the levels of cholesterol-modified hedgehog ligand
Wierbowski et al. (2020)	Dispatched and scube mediate the secretion of the cholesterol-modified hedgehog
Cannac et al. (2020)	Dispatched dedicates to the release of cholesterol-modified hedgehog
Rohatgi et al. (2009)	Normal sterol concentrations are required for proper activation of Smoothened
Byrne et al. (2016)	Oxysterols are allosteric activators of the oncoprotein Smoothened
Huang et al. (2016)	Hedgehog signaling controls Smoothened by regulating its access to cholesterol
Luchetti et al. (2016), Xiao et al. (2017)	Cholesterol influences Hedgehog signaling by directly activating Smoothened
Kowatsch et al. (2019)	The regulation of Smo by Ptc1 is thought to be dependent on cholesterol

## Requirement for Cholesterol in Hedgehog Pathway Transduction

The HH signaling mainly comprises of four parts, as depicted in Figure 2, including HH ligands from the HH secreting cell, transmembrane receptors, the GLI (Glioma-associated oncogene) transcriptional factor family of zinc-finger DNA-binding proteins, and the downstream target genes (Carballo et al., 2018; Cortes et al., 2019). In the absence of HH ligand, PTCH1, a twelve-transmembrane-span receptor, inhibits the activity of SMO, a member of the Frizzled family of seven-pass transmembrane receptor-like proteins. Binding of HH to receptor PTCH1, which is promoted by the membrane co-receptor proteins CDO (CAM-related/down regulated by oncogenes), BOC (brother of CDO) and GAS1 (growth arrest-specific 1) (Kim H. et al., 2020; Echevarría-Andino and Allen, 2020; Kim Y. et al., 2020), leads to inactivation of PTCH1 and activation of SMO with its release from the inhibitory effect by PTCH1. Intriguingly, recent biochemical, structural and cell biological studies have demonstrated that cholesterol also acts as a second messenger to communicate HH signals between PTCH1 and SMO (Table 2). PTCH1 could move cholesterol from the outer to the inner leaflet of the membrane in exchange for potassium ion export (Kinnebrew et al., 2021). A study on the structural analysis of cholesterol-activated *Xenopus laevis* SMO (xSMO) indicated that cholesterol-mediated SMO activation is correlated with the rearrangement of CRD orientation and a cation-lock opening in the transmembrane domain (TMD) of SMO (Huang et al., 2018). A large number of signaling researches and molecular dynamics simulations analyze the structural basis for PTCH1-SMO regulation (Myers et al., 2017; Zhang et al., 2018; Kinnebrew et al., 2019; Radhakrishnan et al., 2020). For example, PTCH1 restricts the access of cholesterol to SMO by controlling the flipping of cholesterol in PM lipid bilayers. Specifically, PTCH1 limits the cholesterol content in the inner leaflet, which may affect cholesterol level in SMO-containing compartments (Zhang et al., 2018). Although the exact mechanisms remain largely vague, studies carried out in the past few years suggest that SMO exists in active and inactive conformational states and PTCH1 regulates the sub-cellular localization of SMO as well as several progressive phosphorylation sites in SMO (Byrne et al., 2018).

**TABLE 2:** Summary of cholesterol functions in PTCH1 and SMO.

References	Nature of role
Kinnebrew et al. (2021)	PTCH1 moves cholesterol from the outer to the inner leaflet of the membrane in exchange for potassium ion export
Huang et al. (2018)	Cholesterol mediated Smo activation involves the rearrangement of CRD orientation and a cation-lock opening in the TMD.
Byrne et al. (2016)	Cholesterol was proposed as an endogenous SMO ligand capable of regulating Hh signaling engages the CRD groove
Zhang et al. (2018)	PTCH1 restricts the access of cholesterol to SMO by controlling the flipping of cholesterol in plasma membrane lipid bilayers.
Hu and Song, (2019)	PTCH1 control the accessibility or enzymatic activity of unknown protein(s) responsible for cholesterol modification of Smoothened
Kinnebrew et al. (2019)	PTCH1 inhibits SMO by reducing accessible cholesterol from the ciliary membrane
Radhakrishnan et al. (2020)	PTCH1 functions as a membrane remodeling machine to inhibit SMO by changing the cholesterol composition of the ciliary membrane
Myers et al. (2017)	Regulation of SMO by protecting its 7TM domain from cholesterol, or by providing an inhibitor that blocks this cholesterol activation

## Insights From Diseases

As an essential sterol found in vertebrates, cholesterol plays a pivotal role in regulating normal functions of cells, tissues and organs, such as acting as a precursor for steroid hormone biosynthesis, regulating the fluidity and permeability of cell membranes and as well as playing roles as a covalently modifying protein (Porter et al., 1996; Miller, 2017; Xiao et al., 2017; Cole et al., 2019). During the early embryonic development, cholesterol plays an essential role in controlling the activity and function of HH signaling pathway, and without sufficient cholesterol, HH signaling works aberrantly, resulting in abnormal development and relevant syndromes. For example, Emopamil binding protein (EBP, also known as 3-beta-hydroxysteroid-Delta (Onodera et al., 2020), Delta(7)-isomerase) mutation-associated is caused by abnormal cholesterylation of SMO (Qiu et al., 2021). Patients with CDPX2 are characterized by cartilage abnormalities around vertebral column, pelvis and long bones (Qiu et al., 2021). Over the past 30 years, progresses in the field of etiology have emphasized that a large amount of developmental malformations of the syndromes caused by abnormal HH signaling transduction are due to the defects of cholesterol biosynthesis, such as holoprosencephaly, a severe brain malformation. Interestingly, it is recently found in mice that inactivation of *SHH* causes axial patterning defects with a phenotype of holoprosencephalic, which is similar to that found in the mice with deficiency of *Megalin* and in some of the human patients with Smith–Lemli–Opitz syndrome (SLOS) as well. In addition, by using chick embryos some studies also confirmed that treatment of cyclodextrin, a cyclic oligosaccharide that forms non-covalent complexes with sterols and can be thereby used to extract and deplete cholesterol from living cells, causes variable loss of the frontonasal process and other midline structures in chick embryos, and the spectrum of facial defects is similar to that resulted from the exposure to HH-pathway antagonist jervine (DeSesso, 2020). Likewise, mutation of *SHH* gene in human causes the similar phenotype as that has been shown in the patients of SLOS. It can be thereby confirmed that at least three ways in HH signaling transduction are associated with metabolism of cholesterol, including the covalent modification of HH in the process of HH maturation and secretion, the action of PTCH1 with putative SSD (Gelissen and Brown, 2017), and the cholesterylation of SMO.

## Hedgehog Signaling Regulates Cholesterol

Since its discovery nearly 30 years ago, the essential role of HH signaling in embryonic development and cancer progress has been clarified, and several mechanisms by which cholesterol regulates HH signaling have been investigated, whereas there was no evidence until 2015 showing HH mutually affects cholesterol (Tang et al., 2015b). Hence, it is interesting to determine whether cholesterol is also regulated by HH signaling, which would further reflect the association of HH with process of other metabolic diseases. HH signaling plays a central role in the development of many steroidogenic tissues, such as the ovary, the adrenal cortex, the testis and the placenta, suggesting HH signaling is involved in the regulation of lipid metabolisms such as steroidogenesis (shown in Table 3) (Tang et al., 2015b; Finco et al., 2015; Matz-Soja et al., 2016; Zhu et al., 2016; Rennert et al., 2017; Albert et al., 2018; Finco et al., 2018; Takai et al., 2019; Luo et al., 2020). The abnormality of HH pathway is also identified to be involved in hepatic steatosis. A previous study utilizing transgenic mice with liver-specific deletion of *SMO* shows that inhibition of HH pathway in hepatocytes causes steatosis by altering the expression of transcription factors GLI1 and GLI3 (Matz-Soja et al., 2016). In contrast, activation of HH signaling reverses the GLI-code and alleviates hepatic steatosis. Additionally, Rennert *et al.* reported that female mice with downregulation of embryonic and hepatic HH signaling results in an increase of testosterone levels, and infertility characterized by reproductive organs and acyclicity, indicating the impaired hormonal balance in the liver *via* the abnormal induction of steroidogenesis (Rennert et al., 2017).

**TABLE 3:** Summary of role for HH in steroid-associated organs and steroid metabolisms.

References	Nature of role
Tang et al. (2015b)	The activation of Hh signaling triggers conversion of cholesterol to progesterone and estradiol
Finco et al. (2015)	Hedgehog signaling pathway is important in steroidogenesis of endocrine tissues
Finco et al. (2018)	Excessive activation of Hh signal affects vascular development in mice
Albert et al. (2018)	The SHH pathway proteins show particular spatiotemporal expression patterns during adrenals development
Takai et al. (2019)	Hh signalling is required for maintenance of somatic cyst stem cells in the Drosophila testis
Zhu et al. (2016)	Shh/Gli2 and Shh/Gli3 signals are required for murine placentas development
Matz-Soja et al. (2016)	HH signaling is crucial for the human pregnancy maintenance and fetal development
Rennert et al. (2017)	The Hh pathway plays a pivotal role in hepatic steatosis
Luo et al. (2020)	The hepatocyte-specific deletion of Smo induces steroidogenesis and results in an impaired hormonal balance

**FIGURE 3:**
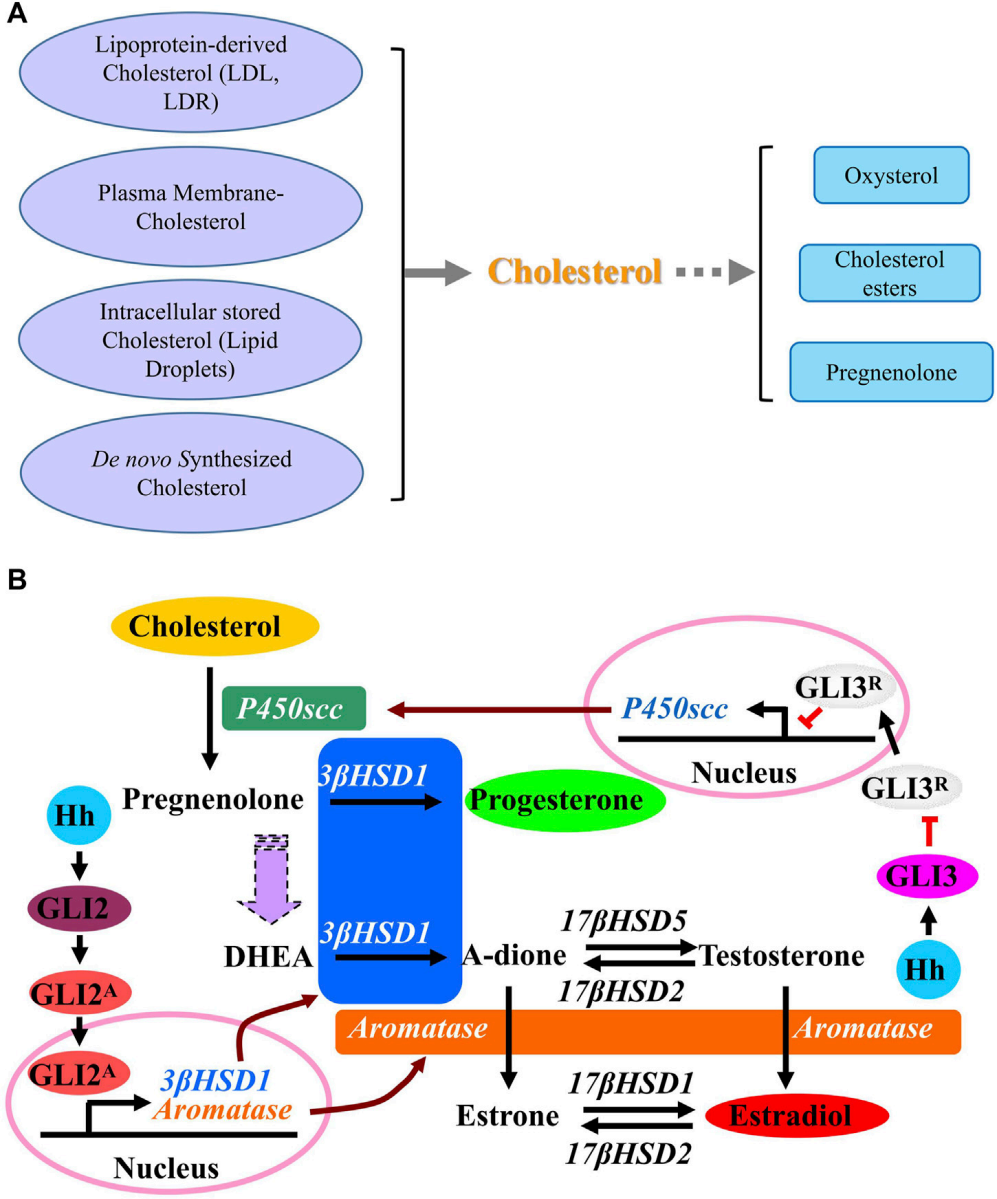
Cholesterol synthesis pathways. **(A)** A schematic description showing the sources of cholesterol and the products of cholesterol. **(B)** A schematic description of effect of Hedgehog signaling on metabolism of cholesterol. Activation of Hh signaling induces the conversion of cholesterol to progesterone (P4) and estradiol (E2) through up-regulating the expression of steroidogenic enzymes including P450scc, 3β-HSD1 and aromatase. GLI3 is required for Hh-induced P450scc expression, while GLI2 mediates the induction of 3β-HSD1and aromatase. Abbreviations: P450scc, P450 cholesterol side chain cleavage enzyme; 3β-HSD1, 3β-hydroxysteroid dehydrogenase type 1; 17β-HSD1, 17β-hydroxysteroid dehydrogenase type 1; 17β-HSD2, 17β-hydroxysteroid dehydrogenase type 2; 17β-HSD5, 17β-hydroxysteroid dehydrogenase type 5; DHEA, Dehydroepiandrosterone.

There are multiple steps in steroidogenesis from cholesterol with the first step of cellular allocation of cholesterol, and cholesterol is recognized to be the common original material for biosynthesis of all kinds of steroid hormones in cells (Luo et al., 2020; Xu et al., 2020; Schade et al., 2020; Guo et al., 2018). In cells many sources of cholesterol can be made available for steroidogenesis, including cholesterol that is delivered by serum low-density lipoprotein (LDL) and high-density lipoprotein (HDL), cholesterol that is internalized from the plasma membrane, hydrolysis of intracellular cholesterol esters (CE) stored in lipid droplets and as well as *de novo* synthesis (Figure 3A) (Luo et al., 2020). *De novo* synthesis of cholesterol in cells is from acetyl-CoA and acetoacetyl-CoA in the ER through several stepwise enzymatic reactions. First, in combination with acetoacetyl-CoA, acetyl-CoA is converted to 3-hydroxy-3-methylglutaryl-CoA (HMG-CoA) in the cytosol. Then, as the rate-limiting enzyme of the pathway, hydroxy-methylglutaryl-CoA reductase (HMG-CoAR) converts HMG-CoA to mevalonate. HMG-CoAR contains a conserved SSD, which is characterized by five membrane-spanning α-helices (Finco et al., 2015; Guo et al., 2018; Wangeline and Hampton, 2020) and is necessary for its attachment to the protein Insig on ER membrane as well as for the regulation of its degradation (Theesfeld and Hampton, 2013; Zhou et al., 2020). In the following steps, mevalonate is converted successively to isopentenyl pyrophosphate, squalene and lanosterol. Finally, lanosterol undergoes a series of 19-additional reactions to produce cholesterol. Some products from cholesterol have been discovered, such as oxysterol and cholesterol esters, and once cholesterol is successfully transferred into the mitochondria, it is catalyzed by P450 cholesterol side-chain cleavage enzyme (P450scc) to produce pregnenolone in mitochondria (Bose et al., 2021). Then pregnenolone moves from the mitochondria to the ER where following reactions happen with the results of production of different hormones (Finco et al., 2015).

A previous report reveals the novel function of HH that HH acts as a regulator in stimulating the conversion of cholesterol to steroid. Activation of HH signaling induces the conversion of cholesterol to progesterone and estradiol through up-regulating the expression of steroidogenic enzymes including P450scc, 3β-hydroxysteroid dehydrogenase type 1 (3β-HSD1) and aromatase. As shown in Figure 3B, while GLI3 is required for HH-induced P450scc expression, GLI2 mediates the induction of 3β-HSD1 and aromatase. As a novel role of HH signaling in regulating the metabolism of cholesterol with up-regulated expression of genes *P450scc*, *3β-HSD1* and *aromatase*, which are essential for steroidogenesis during human pregnancy, it is likely that HH signaling is also essential for human pregnancy maintenance, since a successful pregnancy requires steroidogenesis from cholesterol in placenta. Later in 2016 and 2018, Zhu *et al.* and Raleigh *et al.* discovered that GLI up-regulates the expression of oxysterol synthase HSD11β2, and HSD11β2 generates oxysterols that are associated with the cellular cilia and bind to SMO, which leads to the activation of SMO and promotion of HH pathway activity (Zhu et al., 2016; Raleigh et al., 2018), suggesting that the inhibition of SMO-activating oxysterol production may be therapeutically useful for patients with HH-associated diseases.

Given that HH signaling regulates chondrocyte differentiation and is activated in human and murine osteoarthritis, recent study discovers that HH signaling regulates cholesterol homeostasis in osteoarthritis using human osteoarthritis samples by up-regulating genes that govern cholesterol homeostasis, which alters cholesterol accumulation in chondrocytes (Ali et al., 2016). Moreover, chondrocyte-specific cholesterol accumulation causes an osteoarthritis phenotype in genetically modified mice, while reducing cholesterol accumulation attenuates the severity of osteoarthritis in mice *in vivo* and decreases expression of proteases in human cartilage *in vitro*.

## Perspective

The HH signaling that was first identified in *Drosophila* a few decades ago, has been clarified to be involved in the development of both vertebrates and invertebrates and in the progress of cancer. Owing to rapid progress over the last several years, we have a better understanding of the mechanisms by which HH signaling is regulated by cholesterol, and recent work further uncovers the mechanisms by which HH affects cholesterol regulation. Advances in our knowledge of the cross-talk between HH signaling and cholesterol provides a framework which can be used to illustrate unknown mechanisms of clinical diseases, and further fulfill the biological significance of HH signaling to the known mechanisms. Aberrant HH signaling can lead to abnormal lipid homeostasis, which affects atherosclerosis and hepatosteatosis. Further research, especially progress in metabolomics and genetic studies will help reveal novel and intricate details of the biochemical and molecular cellular biological aspects of HH signaling. Currently, there are still some challenges in this area. What is the specific mechanism by which sterol participates in the regulation of SMO by PTCH1? How exactly does cholesterol modification of SMO activate HH signaling and transduce downstream events? How aberrant cholesterol metabolism affects Hh signaling and leads to different clinical manifestations? Deeper investigations in this field will potentially benefit disease therapy, especially in understanding the relation between HH signaling and related sterol metabolisms, which will provide potential targets in developing novel drugs for relevant diseases in the future.
